# Agricultural Greenhouses Detection in High-Resolution Satellite Images Based on Convolutional Neural Networks: Comparison of Faster R-CNN, YOLO v3 and SSD

**DOI:** 10.3390/s20174938

**Published:** 2020-08-31

**Authors:** Min Li, Zhijie Zhang, Liping Lei, Xiaofan Wang, Xudong Guo

**Affiliations:** 1 Key Laboratory of Digital Earth Science, Aerospace Information Research Institute, Chinese Academy of Sciences, Beijing 100094, China; limin2018@radi.ac.cn (M.L.); leilp@aircas.ac.cn (L.L.); 2 College of Resources and Environment, University of Chinese Academy of Sciences, Beijing 100190, China; 3 Key Laboratory of Land Use, Ministry of Natural Resources, China Land Surveying and Planning Institute, Beijing 100035, China; wangxf.10s@igsnrr.ac.cn (X.W.); xudongguo@hotmail.com (X.G.)

**Keywords:** agricultural greenhouse detection, convolutional neural network, Faster R-CNN, YOLO v3, SSD

## Abstract

Agricultural greenhouses (AGs) are an important facility for the development of modern agriculture. Accurately and effectively detecting AGs is a necessity for the strategic planning of modern agriculture. With the advent of deep learning algorithms, various convolutional neural network (CNN)-based models have been proposed for object detection with high spatial resolution images. In this paper, we conducted a comparative assessment of the three well-established CNN-based models, which are Faster R-CNN, You Look Only Once-v3 (YOLO v3), and Single Shot Multi-Box Detector (SSD) for detecting AGs. The transfer learning and fine-tuning approaches were implemented to train models. Accuracy and efficiency evaluation results show that YOLO v3 achieved the best performance according to the average precision (mAP), frames per second (FPS) metrics and visual inspection. The SSD demonstrated an advantage in detection speed with an FPS twice higher than Faster R-CNN, although their mAP is close on the test set. The trained models were also applied to two independent test sets, which proved that these models have a certain transability and the higher resolution images are significant for accuracy improvement. Our study suggests YOLO v3 with superiorities in both accuracy and computational efficiency can be applied to detect AGs using high-resolution satellite images operationally.

## 1. Introduction

Agricultural greenhouses (AGs) are an important technique in modern agriculture to satisfy the human demand for farm products [1,2,3,4]. In recent years, AGs have been expanding sharply and changing the agricultural landscape in many regions, especially in China, Europe, North Africa, and the Middle East [5]. The vigorous construction and expansion of AGs, however, has induced many issues in land management, such as occupied high-quality cultivated land [6], damaged soil [7], pollution of plastic wastes [4] etc. To address these problems, we need an effective detection method to monitor the spatial distribution of AGs, in order to reasonably develop AGs and protect the cultivated land [8].

The remote sensing images from satellite, airborne, and unmanned aerial vehicles (UAV) have been enabled to provide a significant contribution to detecting AGs, since AGs detection became annual routine work for the competent department in China, such as the Ministry of Natural Resources and the Ministry of Agriculture and Rural Affairs. However, features of remotely sensed images could vary under different observing conditions of atmosphere, sensor quality, solar illumination, and peripheral surroundings. Moreover, different materials with varying thickness, reflection and transmission properties were used in AGs coverings, presenting a highly fragmentized and heterogeneous appearance of AGs in remote sensing images. As a result, AGs detection was mainly achieved by visual interpretation with remote sensing images in government management. This method, depending on the level of expertise, is time-consuming and far from a large-scale automatic AGs detection requirement. 

The recent advances in pattern recognition and machine learning [9,10] have given great opportunities for automatic information extraction based on big data [11], including high spatial resolution remote sensing images [12]. This is largely driven by the wave of deep learning [13], which describes the most representative and discriminative features by multi-layer neural networks in a hierarchical manner. During the past decades, deep learning has made a considerable breakthrough, not only in natural language processing fields [14,15], but also in computer vision tasks and other applications [16,17]. Convolutional neural networks (CNNs), as one of the most successful network architectures in deep learning methods through end-to-end learning, have gradually replaced the conventionally featured engineering in image analysis due to their superiority in higher-level feature representation. Consequently, CNNs have been widely introduced to geoscience and remote sensing communities, such as target extraction [18,19,20,21,22], land classification [23,24], and object detection tasks [25,26,27].

CNN-based object detection algorithms can currently be summed up into two categories. One is represented by regions with CNN (R-CNN) and its improved methods [28,29,30], named two-stage detectors. These approaches firstly generate a series of sparse candidate boxes based on region proposal algorithms, then apply a CNN detector to perform bounding box regression and classification. The other type of algorithms, named single-stage detectors, predict bounding boxes and class probabilities of targets from full images synchronously, which is represented by Single Shot Multi-Box Detector (SSD) [31] and You Only Look Once (YOLO) series [32,33,34] algorithms. These algorithms derived from CNN have achieved great success in major competitions, such as PASCAL VOC (Pattern Analysis, Statistical Modelling and Computational Learning Visual Object Classes) [35], ImageNet [36], and Coco (Common Objects in Context) [37], where objects were detected from natural images.

As the current deep learning is data-driven, the performance of object detection algorithms, e.g., the accuracy and efficiency, show large variations depending on the data source. Remote sensing images obtained from satellite sensors are much more complex than natural images, since interference from the atmosphere, background, incidence angle, and illumination is inevitable. Additionally, the size of the targets in satellite images is relatively smaller than that in natural images due to the influence of the sensor’s spatial resolution, increasing the difficulty of remotely sensed object recognition. As a result, most studies implemented CNN-based object detection on limited open datasets and different private datasets. For instance, Cheng et al. created and made an open, high-quality data set named NWPU VHR-10 with large-size training samples from remote sensing images [38]. This dataset contains a total of 3775 object instances related to 10 geospatial object classes, and has been widely used in the earth observation community [39]. Zhang et al. adopted an improved Faster R-CNN to detect ships from high-resolution optical remote sensing images and achieved a higher recall and accuracy compared with SSD and YOLO v2 [40]. Chen et al. conducted end-to-end trainable airplane detection on Google Earth images by using SSD architecture and achieved 96.23% average precision [41]. Ma et al. worked on the detection of collapsed buildings in post-earthquake remote sensing images based on the improved YOLO v3, which provided a 90.89% precision [42]. 

There are few relevant studies that evaluate the generalization of CNN-based object detection methods for agricultural applications due to the lack of training samples and the complex properties of landscapes. In this research, we conducted a comparative evaluation of three well-established deep learning algorithms, which are Faster R-CNN, YOLO v3—the third version of the YOLO [43], and SSD, to detect AGs from multi-source satellite images. To increase the detection accuracy under the limitations of training samples, a pre-trained network was introduced as a base and the transfer learning approach was adopted. The main objectives of this research are as follows:

- Investigating a desirable method for AG detection that can provide accurate and effective information of AGs for governmental management.
- Introducing the transfer learning and fine-tuning approaches to improve the performance of CNN-based methods on agricultural applications.
- Performing an evaluation on two independent test sets to investigate the transferability of the models.

## 2. Data and Methods

In this section, the used satellite images and sample preparation are given first. Next, we elaborate on the theory of the three network architectures. Lastly, hyper-parameters and training processes are introduced.

### 2.1. Data

The dataset was collected at 113°46′–116°20′ E and 38°14′–40°00′ N, within the city of Baoding, Hebei province, China. It is a major area for agricultural production, where AGs have been greatly developed in the last few decades. According to our field survey, transparent plastic is the most widespread material used for AG roofing. In addition, AGs made of glass fibers and ethylene-vinyl acetate copolymer (EVA) are also prevalent in this area.

**Figure 1:**
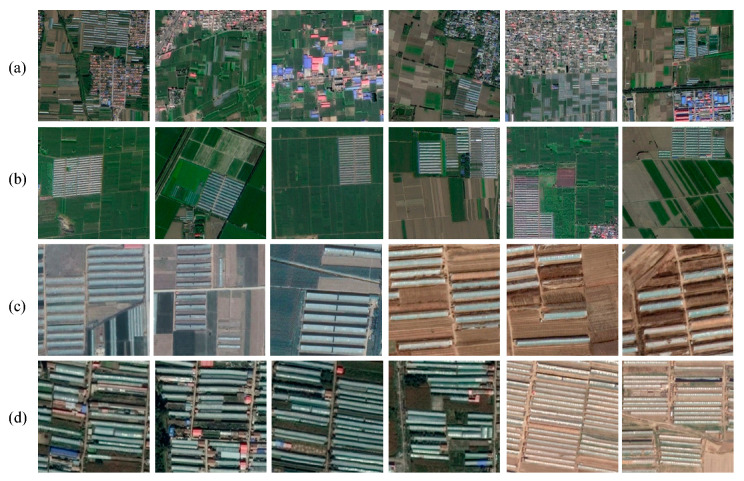
Examples of image tiles from the regions of interest (ROIs): (**a**) scatted agricultural greenhouses (AGs) surrounded by complicated backgrounds in GF-1 images; (**b**) regular AGs surrounded by apparent backgrounds in GF-1 images; (**c**) individual AGs surrounded by simple backgrounds in Gaofen-2 (GF-2) images; (**d**) fragmented and serried AGs in GF-2 image.

The AGs dataset produced in this area were from available remote sensing images taken by the Gaofen-2 (GF-2) satellite, as Gaofen series satellites are the most important data source for governmental land use planning in China. The spatial resolution of GF-2 images is 1 m after image fusion. In addition, the Goafen-1 (GF-1) satellite images with a spatial resolution of 2 m in the same geographical area were introduced as supplementary data. GF-1 has three spatial resolutions in the panchromatic band (2 m) and multispectral band (8 m/16 m), and its repeat is 41 days. In this study, we fused GF-1 data into 2 m multispectral data and processed together with GF-2. This is mainly designed to increase the diversity of samples and explore the transferability of these methods on different data sources but with similar AG styles. All of these images, containing various orientations, aspect ratios, and pixel sizes of the objects, were taken with a dynamic range from December 2016 to December 2017 to ensure the diversity of samples. Due to the limited size and computational power, we pre-processed images and extracted areas for regions of interest (ROIs) that may contain AGs, such as crop fields. Then, the ROIs were regularly cropped into multi-scale tiles (300 × 300, 416 × 416, 500 × 500, 800 × 800, 1000 × 1000) without overlap, which is essential for correcting prediction results of both coarse-scale and fine-scale detail in the images [44]. Referring to the format of the PASCAL VOC dataset [36], we carefully annotated these images in the same manner and saved annotations as XML (extensible markup language) files. The labeled sample tiles of GF-1 and GF-2 images were 413 and 964, respectively, containing a total of 18,385 target AGs. Figure 1 shows examples of samples under different conditions.

The prepared sample dataset was divided into three parts: training, validation, and test sets. The training set was adopted to train individual detection models and generate proposal regions, while the selection of the optimal hyper-parameters was based on the validation set. The test set was used to evaluate detection results from the model. In this study, the labeled image tiles were randomly assigned to one of the three sets, approximately following the ratio of 60%:20%:20%. In order to enrich the training data and enhance the robustness of the detectors in the training step, we duplicated every tile by random cropping. The specific information about the dataset is presented in Table 1.

### 2.2. Network Framework of Faster R-CNN, YOLO v3 and SSD

As the typical deep learning methods for object detection, Faster R-CNN, YOLO v3, and SSD have been widely used in the study of remote sensing images. We briefly set forth the three network architectures here and Table 2 summarizes the properties of these models.

#### 2.2.1. Faster R-CNN

**Figure 2:**
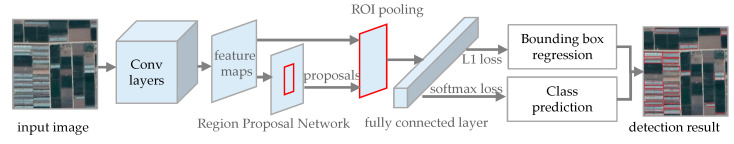
Architecture of Faster R-CNN in detecting AGs.

Figure 2 presents the basic architecture of Faster R-CNN. It consists of two modules: a region proposal network (RPN) and a Fast R-CNN detector. The RPN is a fully convolutional network for proposal generation. Each location of the feature maps can produce 9 anchors of 3 different scales and 3 aspect ratios, and then these anchors are judged as positive or negative based on the availability of targets. The positive and negative anchors are selected randomly by a 1:1 ratio as a minibatch to prevent bias occurring, which are utilized to generate candidate regions by comparing with ground truth boxes of the objects at the process of training. Therefore, after introducing the convolutional feature maps of arbitrary size into the RPN, a batch of feature information can be generated, describing the region proposals that contain the candidate objects or not. The Fast R-CNN detector shares a set of convolutional layers with the RPN. To generate superior object proposals, a base network can be utilized to extract features in these convolutional layers. In this research, we compared the Visual Geometry Group (VGG) [44], and Residual Neural Network (ResNet) for this model, and the results on the validation set proved that the VGG-16 is optimal in this dataset. In the second module, the ROI pooling layer maps the extracted convolutional features of the proposals into uniform-sized feature vectors, which next serve as the input of fully connected layer. Finally, a set of probability values for the different classes can be derived and a softmax classifier is used to determine which category it belongs in, while a regressor is adopted to access the more accurate coordinate values of the bounding boxes.

#### 2.2.2. YOLO v3

**Figure 3:**
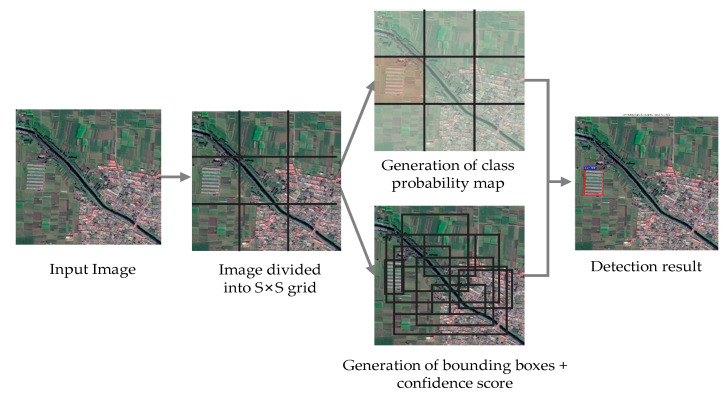
Stages of YOLO v3 model in detecting AGs.

YOLO v3 is both an inheritance and improvement over YOLO v1 [32] and YOLO v2 [33]. All input images in the YOLO v1 algorithm are resampled to a fixed size and divided into S × S grid. A grid cell can only be linked with one object, where a fixed number B of bounding boxes and their corresponding confidence score are directly predicted. At the same time, a sequence of probabilities of the objects in each class is output using the fully connected layer. However, there may be more than one box around the same ground truth target. To eliminate redundant predictions, the non-maximum suppression (NMS) with an intersection over union (IoU) threshold is used to select detection boxes with the highest confidence score. IoU measures the overlap between the predicted and the ground truth bounding boxes. When the calculated IoU of detection boxes is higher than the pre-defined threshold, NMS just maintains the detection box with the highest confidence and discards the others. Thus, an object detection problem is successfully reconstructed to an end-to-end regression task. YOLO v1 significantly outperforms R-CNN variants in terms of detection speed, as the complete image is processed only once. However, it struggles to perform satisfactorily in localization, especially when dealing with small objects. Given these problems of YOLO v1, a second version YOLO v2 was proposed, which introduces batch normalization as well as the anchor box mechanism for prediction. In addition, it removes the fully connected layers of the YOLO v1, turning the entire model into a fully convolutional network. Such modification allows more bounding boxes to be calculated, and input data of arbitrary dimension can be operated on the network. As a result, the accuracy of YOLO v2 shows significant improvements over YOLO v1, although its performance on small objects is still not desirable. The next generation YOLO v3 enhances the detection results further by adopting multi-label classification and featuring pyramid networks. In addition, the YOLO v3 upgrades the substrate network Darknet-19 to Darknet-53 to explore deeper feature information of objects. With this structural design, the YOLO v3 makes up for the deficiency of YOLO v2 and outperforms most of the detection methods. The successive stages of YOLO v3 are demonstrated in Figure 3.

#### 2.2.3. SSD

**Figure 4:**
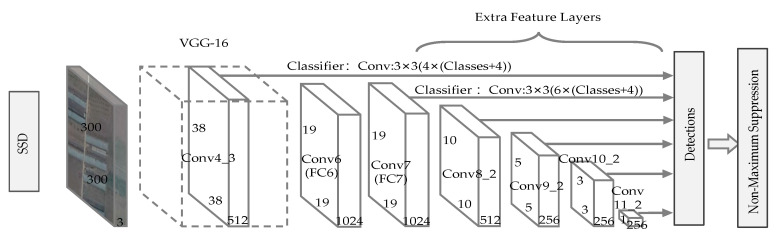
Overall architecture of SSD.

The overall architecture of SSD is illustrated in Figure 4. The SSD algorithm combines the anchor box mechanism of Faster R-CNN and the regression idea of YOLO v1. The first few layers are a commonly used architecture in object detection models, which is called the base network. Here, we adopt the same VGG-16 network as Faster R-CNN. Moreover, SSD adopts a pyramid structure, namely multi-dimensional feature maps after the base network. As the spatial resolution of feature maps keeps decreasing, the spatial information of the detail is continuously lost while abstract semantic features are growing. As a result, small objects and large objects can be detected at the same time by features of different depth, which is important for solving the changes in object scales. 

Using the concept of anchor box in Faster R-CNN as a source of reference, SSD establishes a group of default boxes with different scales and aspect ratios at each pixel of feature maps and serves them as the benchmark to generate predicted bounding boxes. Generally, the default boxes in different feature maps are different sizes, and default boxes of different scales and aspect ratios are set in the identical feature map. These boxes are competent to encompass objects that are of various shapes and sizes. In the initial process of training, the default box is matched with ground truth boxes, aiming to find a specific default box with the largest IoU for each ground truth object. Then, the remaining default boxes are matched with any ground truth box, and assigned as negative samples if the IoU of the two is less than the threshold (set as 0.5 in this work [36]). Instead of using all negative samples for prediction, the confidence score for each default box is calculated and the three with the highest score were selected in this study. Thus, we successfully controlled the ratio of positive samples and negative samples at 1:3, which facilitates the learning process and allows faster optimization. As in the YOLO, the final predictions are proposed after the NMS algorithm is applied.

### 2.3. Model Training

In this study, all the architectures were run on a workstation with two TITAN RTX GPU (2 × 8 GB) and implemented with the PyTorch open-source deep learning framework, which was developed by the Facebook team. The operating system was Ubuntu 16.04 LTS. To ensure that comparison was conducted under fair conditions, we need to convert the YOLO v3 darknet model to the PyTorch framework by modifying darknet weights and model to corresponding compositions of PyTorch.

Transfer learning is a very important and effective strategy in deep learning, which focuses on gathering useful information from a previously trained network and applying it to different but related problems [45]. ImageNet is a natural image dataset for object detection. Most of the base networks are trained on it, because features such as edges, colors, and shapes can be implemented, which form the basis of version tasks. Thus, we introduced pre-trained models to this dataset to initialize the weights and bias through the transfer learning approach. Additionally, the fine-tuning of parameters was performed with our collected dataset to improve the performance as much as possible.

Each detector requires a set of hyper-parameters that need to be configured before training. We started with the default values and then repeatedly trained many times by changing one or more major hyper-parameters. Finally, models with converged loss values and optimal performance on the validation set were selected for our research. 

All three types of CNNs were trained using the stochastic gradient descent (SGD) algorithm. The input image sizes used to train Faster R-CNN and SSD were 512 × 512 and 300 × 300, respectively. For Faster R-CNN, the initial learning rate was 0.01 and the batch size was defined as 8. The training process continued for 30 epochs by reducing the learning rate to half after 20 epochs. One epoch means the feed forward and back propagation processes were completed for the whole training set. For YOLO v3, the batch size was defined as 16. The training loss got converged and showed optimal performance on the validation set until 150 epochs, and the learning rate was 0.0005 with a decay factor of 0.5 for every 30 epochs. For the SSD network, the batch size was defined as 16. The whole training continued for 80,000 iterations with a 0.0001 initial learning rate and a 0.5 decay factor for every 20,000 iterations. The training duration of the Faster R-CNN, YOLO v3, and SSD networks were comparable when the validation accuracy reached a plateau, which took about 4.5 h, 5 h, and 5.5 h, respectively.

## 3. Results and Discussion

### 3.1. Evaluation Metrics

We adopted three widely used measures, which mainly include precision-recall curve (PRC), mean average precision (mAP), and frames per second (FPS), to evaluate the performance of different object detection models. The detailed description of these metrics is introduced as follows.

(a) Precision-recall curve (PRC) is composed of precision (P) and recall (R). It is a more conventional and objective judgment criterion in the field of object detection compared with individual precision or recall metric. The precision measures the fraction of correctly identified positives and detection results, while the recall measures the fraction of correctly identified positives and the total number of all ground truth samples. The precision and recall indicators are calculated as:
  (1) $$\mathrm{precision}=\frac{\mathrm{TP}}{\mathrm{TP}+\mathrm{FP}}$$
  (2) $$\mathrm{recall}=\frac{\mathrm{TP}}{\mathrm{TP}+\mathrm{FN}}$$
  where true positive (TP) indicates the total number of targets successfully detected by the model; false negative (FN) indicates the total number of targets that are falsely identified as other objects; false positive (FP) indicates the total number of predictions that identified other objects as targets.
(b) Average precision (AP) is measured by the area under the PRC for each class. We use the mAP over all classes to evaluate the performance of the model. Since there are only two classes considered, namely AG and background, the mAP of the whole model is equal to the AP of the AG detection. Generally, the higher the mAP, the better the performance.
(c) Frames per second (FPS) measures the number of images that are processed on the model per second. When running on the steady-state hardware, the larger the FPS, the faster the model detects.

### 3.2. Visual Evaluation

**Figure 5:**
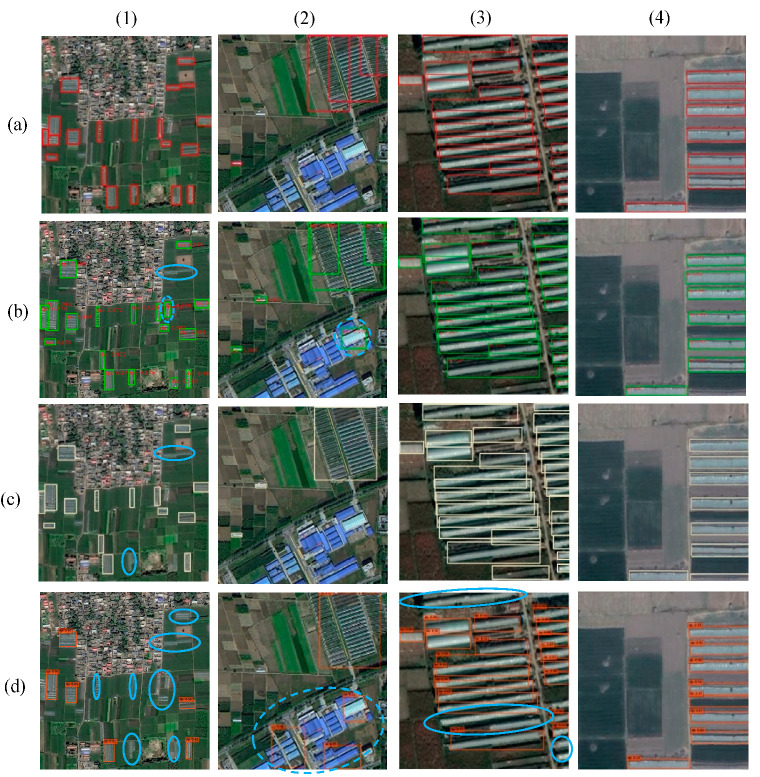
Four subsets (**a**) ground-truth and detection results of different scenarios using (**b**) Faster R-CNN, (**c**) YOLO v3, (**d**) SSD. (**1**) Targets surrounded by crop land and residential areas in GF-1 image; (**2**) targets surrounded by industrial buildings in GF-1 image; (**3**) fragmented and serried targets in GF-2 image; (**4**) simple surroundings and regular targets in GF-2 image. Solid and dotted blue circles represent the existence of missing and erroneous detections, respectively.

In this study, the images of AGs under different scenarios were tested to evaluate the models. Four subsets of ground truth images and their corresponding detection results, obtained by the three models, are presented in Figure 5 with blue circles highlighting incorrect detections. From the perspective of the AGs’ characteristics and the peripheral surroundings, all the three methods have achieved great success in visually detecting AGs that are characterized by distinct geometric shapes and simple background (Figure 5 (4)). The obvious differences in detection accuracy mainly occurred under the following conditions: (1) the individual AGs that are relatively small and scattered around the cultivated land or settlements; (2) industrial buildings that are similar to the AGs in shape and texture; (3) fragmented or serried targets that are situated in contextually mixed areas. 

As we all know, spatial resolution is one of the key factors affecting the detection performance of the model. On the one hand, the AGs varying in shape or size exhibit different scales on the same image. On the other hand, the size of the same AG varies on images with different resolutions. According to the detection results presented in Figure 5, these three models have achieved great recognition on GF-2 images (Figure 5 (3–4)), while their performance notably decreased on the GF-1 set (Figure 5 (1–2)). Specifically, Faster R-CNN and YOLO v3 were completely free of erroneous detections on the higher resolution GF-2 images. Only in areas where AGs are inclined and densely concentrated, SSD made some errors in small target detections. However, all three models had obviously missing and erroneous detections on GF-1 test images, with SSD performing the poorest. This is probably explained by the fact that the input image size of SSD is the smallest, resulting in the largest scaling degree of original images. Since small targets are represented with very few numbers of pixels, less detailed features can be accessed by shallow convolutional networks, while it is more difficult to obtain semantic information from deeper feature maps.

### 3.3. Evaluation with Metrics

**Figure 6:**
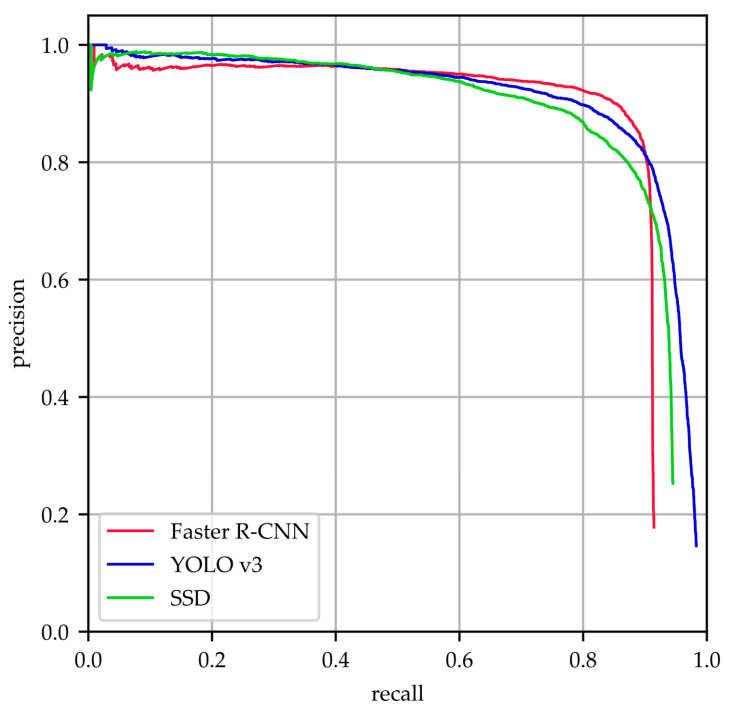
Precision-recall curves (PRCs) for Faster RCNN, YOLO v3 and SSD.

Comparison results of the three models on the integrated test set measured by PRCs are shown in Figure 6. A curve at the top of the PRCs indicates a better performance. We can observe that all the networks provided acceptable performance for AG detection, but it is hard to evaluate which one is the best from the curve. Therefore, we adopted the mAP as a quantitative metric to evaluate the accuracy of the three models.

The mAP was calculated for all networks with a pre-defined IoU threshold. Table 3 shows the numerical comparison results. All of the three models had the mAP above 80% on the integrated test set with diverse AGs, illustrating that these three methods were able to achieve favorable results on this dataset. YOLO v3 demonstrated the highest mAP (90.4%) among the three models. The mAP of Faster R-CNN and SSD were similar, with only a slight difference of 1.1%. A preliminary analysis suggested that the network Darknet-53, a deeper structure than VGG-16, is more competent in extracting diverse and complex features of targets, playing a fundamental role in the detection accuracy improvement of YOLO v3.

Additionally, the models that trained on the integrated training set were further applied to two independent test sets with different resolution images to evaluate the transferability of the models. The experimental results of the mAP are appended to Table 3. Among the three models, YOLO v3 achieved the best performance on these two independent tests. Faster R-CNN and SSD provided comparable mAP on higher resolution GF-2 images, while the accuracy difference was mainly derived from the GF-1 test set. Moreover, it is apparent that all three models showed significantly different detection capabilities between the two test sets. When tested on GF-1 images, the mAP of the three models was no higher than 75% and was about 20% lower than the performance on the integrated test set. By contrast, the minimal mAP of all three models on the GF-2 set was 87.9%, which was much better than the performance on the GF-1 images. On the one hand, it can be attributed to the imbalance between the training samples from the two different data sources. On the other hand, the peripheral surroundings inside the large-scale GF-1 images are more confusing, increasing the difficulties in feature learning. The contrastive results confirmed that these models had a certain degree of transferability and the image resolution is a crucial factor associated with the quality of the detection performance. High-resolution satellite images with relatively apparent characteristics of targets can significantly improve detection accuracy, while low-resolution images will not only lead to difficulties in sample making, but also greatly reduce the quality of the predictions.

The FPS yielded by the three methods is also appended to Table 3. The YOLO v3, with 73 images detected per second, was more than twice as fast as the SSD. The two-stage detector Faster R-CNN, with a FPS of 12, remarkably lagged in comparison to SSD and YOLO v3. Overall speaking, the YOLO v3 showed the best performance among the three detection models if only detection efficiency was considered, while the low detection efficiency of the Faster R-CNN limited its potential applications.

By jointly analyzing the PRC, the mAP metric, and the FPS, it shows that the YOLO v3 exhibited a balanced performance with good localization of objects and a high detection speed. This superiority mainly comes from its inherent strength in the deeper feature extraction network and the single-stage architecture. As a result, YOLO v3 is well equipped to provide faster and more accurate detection of AGs among the three models. 

YOLO v4 [46] is the latest version of the YOLO series network and was developed in April 2020. Given that enhancement of the models can produce breakthroughs in accuracy and efficiency, we also tested YOLO v4 using the integrated dataset in our experiments. The selected network resulted in 91.8% mAP and 98 FPS, which were the highest among all the detectors. These results were expected as the YOLO v4 introduced a new method for data augmentation and modified some existing methods for efficient detection. In this paper, we focus on AG detection for a fair comparison among three typical and comparable approaches. To yield a high accuracy in future applications, more improved and enhanced model architectures should be considered.

## 4. Conclusions

In this paper, we conducted a thorough experimental comparison of three well-established CNN-based object detection models, namely Faster R-CNN, YOLO v3, and SSD, for AG detection from high-resolution satellite images. Their performance, mainly including efficiency and accuracy, was discussed under different scenarios and evaluated with both the integrated test images and independent test sets. The best results were obtained with the YOLO v3 network according to mAP and FPS metrics. The Faster R-CNN also provided promising results with accurate localization. Although SSD provided the worst detection accuracy when the relatively lower resolution images are considered, it was superior in processing time in comparison to Faster R-CNN. The spatial resolution of satellite images is the main factor affecting the detection performance. The higher the spatial resolution, the better the detection quality. Moreover, the transfer learning and fine-tuning on a pre-trained network are effective in providing promising results for object detection from satellite images. In summary, Faster R-CNN and SSD demonstrate certain practicalities in detection accuracy and efficiency individually, but they prove difficult to satisfy the requirements of fast and accurate object detection, simultaneously. YOLO v3 can generally complete fast and accurate AG detection using satellite images in operational monitoring work, which can serve as a basis for governmental land management and decision-making. 

Future research can hopefully improve object detection by making full use of the multispectral and hyperspectral data in satellite images, as the current detection task is usually processed on the color images of RGB composited by satellite sensor bands. In addition, it will also be worthwhile to conduct further research on accessing the footprint, shape, and inclination angle of the AGs while acquiring location and boundary information. Moreover, further studies can plan to generate a large and high-quality dataset from remotely sensed images for the application of agriculture and land management.

## Figures and Tables

**Table 1:** The dataset of samples.

Data Source	Spatial Resolution	Number of Total Samples	Number of Target AGs	Number of Training Samples	Number of Validation Samples	Number of Test Samples
GF-1	2 m	413	14,920	247	83	83
GF-2	1 m	964	3465	578	193	193
Total		1377	18,385	825	276	276

**Table 2:** Comparison of Faster R-CNN, You Look Only Once-v3 (YOLO v3) and Single Shot Multi-Box Detector (SSD).

	Faster R-CNN	YOLO v3	SSD
**Phases**	RPN + Fast R-CNN detector	Concurrent bounding-box regression and classification	Concurrent bounding-box regression and classification
**Neural Network Type**	Fully convolutional	Fully convolutional	Fully convolutional
**Backbone Feature Extractor**	VGG-16 or other feature extractors	Darknet-53 (53 convolutional layers)	VGG-16 or other feature extractors
**Location Detection**	Anchor-based	Anchor-Based	Prior boxes/Default boxes
**Anchor Box**	9 default boxes with different scales and aspect ratios	K-means from coco and VOC, 9 anchors boxes with different size	A fixed number of bounding boxes with different scales and aspect ratios in each feature map
**IoU Thresholds**	Two (at 0.3 and 0.7)	One (at 0.5)	One (at 0.5)
**Loss Function**	Softmax loss for classification; Smooth L1 for regression	Binary cross-entropy loss	Softmax loss for confidence; Smooth L1 Loss for localization
**Input Size**	Conserve the aspect ratio of the original image, and resized dimension ranges from smallest 500 to largest 1000	Random multi-scale input	Resize original images to a fixed size (300 × 300 or 512 × 512)

**Table 3:** Metrics comparison of different models.

	Faster R-CNN	YOLO v3	SSD
mAP (GF-1& GF-2)	86.0%	90.4%	84.9%
mAP (GF-1)	64.0%	73.0%	60.9%
mAP (GF-2)	88.3%	93.2%	87.9%
FPS	12	73	35
